# Understanding the Drawbacks of the Current Tumor Staging Systems: How to Improve?

**DOI:** 10.3390/cancers15041242

**Published:** 2023-02-15

**Authors:** Luca Giacomelli, Rodolfo Sacco, Simonetta Papa, Brian I. Carr

**Affiliations:** 1 Department of Surgical Sciences and Integrated Diagnostics, University of Genoa, 16132 Genova, Italy; 2 Polistudium Srl, 20135 Milan, Italy; 3 Gastroenterology and Endoscopy Unit, Department of Surgical and Medical Sciences, University of Foggia, 71100 Foggia, Italy; 4 Department of Liver Cancer Biology, Liver Transplant Institute, Inonu University, Malatya 44280, Turkey

Tumor stage definition is required for the description of the diagnosis and the development and use of treatment guidelines, as well as to enable clinical research (including clinical trials) and cancer surveillance [1]. However, the staging protocols often present some pitfalls and controversial issues, especially due to continuing advances in molecular oncology approaches and further progress in prognostic classification tools. This requires constant revision of the staging system and modification of guidelines to enable proper patient management and treatment allocation. Nevertheless, in clinical practice, this process is slower than the progress in molecular biology. 

A paradigmatic example of the controversial applicability of tumor staging classification for determining treatment strategies is in the daily management of patients with hepatocellular carcinoma (HCC) [2]. In managing HCC patients, the widely-used "stage hierarchy" approach connects each disease stage to a specific treatment [3]. This intellectual conception underpins the Barcelona Clinic Liver Cancer (BCLC) model, which represents the main example of the application of this strategy [4,5]. In its 20-year history, the BCLC classification has undergone several refinements based on improvements and emerging evidence in HCC management. However, the central idea of recommending a stage-specific therapy has been retained to date [6].

When ‘ideal patients’ for a specific treatment are selected through a treatment algorithm, a large proportion of subjects for whom that therapy could be used do not meet the selection criteria. Concerning HCC, examples include certain intermediate-stage HCC patients for whom the adoption of the extended criteria for a liver transplant or downstaging procedures by transplant centers has allowed access to this kind of therapy [7,8,9]. The BCLC indications have been challenged by several studies showing that patients given potentially higher efficiency treatments than the BCLC standard of care exhibited better outcomes than those treated according to the BCLC algorithm; moreover, treatment modality was an independent predictor of survival within each BCLC-defined stage [10,11,12,13,14]. The general interpretation of the BCLC recommendations has been updated based on the concepts of ‘treatment stage migration’ and ‘treatment stage alternative’, the latter providing further therapeutic options for each BCLC stage [15,16,17]. Another potential proposed strategy involves considering the treatment decision dictated hierarchically by the effectiveness of each therapy, with complete or partial independence from the tumor stage (“therapeutic hierarchy”) [18]. All three conceptual strategies aim to significantly increase adherence to treatment guidelines. 

The experience of the treatment allocation in HCC patient management highlights how a simple stage-linked treatment strategy may not be the best option, especially when personalized, evidence-based approaches emerge and advance quickly, making it difficult to keep classification systems and, consequently, diagnostics and treatment up to date [19]. 

Similarly, current breast cancer staging and classification systems present some pitfalls, as breast cancers with virtually identical TNM characteristics may exhibit highly contrasting behaviors due to divergent molecular profiles [20]. This further highlights the need for alternative approaches based on predictive models with histopathological and molecular predictors, allowing the development of more accessible decision-making algorithms. These would consider traditional non-genomic systems, but also new perspectives on molecular medicine, genetics, and genomics that better explain the heterogeneity of this complex group of diseases.

In light of this evidence, there is a need to engage in a critical discussion of the viability and reliability of the current tumor staging systems of several neoplasms and to provide new knowledge upon which to base additional or alternative prognostic and therapeutic strategies. 

This Special Issue offers a series of five original articles, presented by international leaders, discussing some advances in defining the diagnostic and prognostic significance of various factors and taking into account the increased complexity resulting from the striking advances in diagnostic and prognostic techniques [21,22,23,24,25].

A study by Piórek and collaborators examined the prognostic significance of TNM in patients with a primary tracheal tumor. The authors proposed a simple classification according to TNM to distinguish groups of patients with favorable prognoses and identify patient groups by treatment intent [21]. The pilot study by Macrini and collaborators evaluated a simplified diagnostic model to identify potentially lethal prostate cancer (PC) cases at initial diagnosis. They found that a cribriform pattern/intraductal carcinoma might be a marker of potentially lethal PC. They also suggested that the high incidence of *TP53* and *BRCA2* mutations in de novo metastatic castration-sensitive PC may also have therapeutic implications [22]. Righi et al provided a metabolomic analysis of actinic keratosis and squamous cell carcinoma, suggesting a grade-independent model of squamous cancerization and supporting the expanding notion that all actinic keratoses should be treated independently from their clinical appearance or histological grade because they may be associated with squamous cell carcinoma [23]. Carr and collaborators examined multiple clinical characteristics of patients with HCC and their relationship to death. They created a three-parameter tool (comprising portal vein thrombosis, tumor numbers - multifocality -, and blood alpha-fetoprotein levels) to examine the characteristics and survival of patients with normal and abnormal levels of this tool. They found that patients with large tumors and normal levels of these three parameters were associated with longer survival than any group including patients with portal vein thrombosis [24]. Lastly, the meta-analysis by Facciorusso and collaborators compared microwave ablation with radiofrequency ablation for the treatment of HCC in terms of efficacy and safety, suggesting similar outcomes for the two techniques [25].

In conclusion, the available data suggest that establishing a more accurate cancer staging system is required to enhance patient management and to define more effective treatment algorithms and meaningful scientific research. At the same time, the available evidence raises the question of whether it will be possible to identify therapeutic targets and individualize treatment based on the predictive value of new molecular markers, or whether histopathological and anatomical systems, constantly being updated, should continue to be the basis of staging systems.
